# Supplementary material for: Predictors for Early and Late Death in Adult Patients with COVID-19: A Cohort Study
Source: Int J Environ Res Public Health. 2022 Mar 12;19(6):3357. doi: 10.3390/ijerph19063357 (PMC8954087; doi:10.3390/ijerph19063357)
Supplement: Supplementary file 1 [file ijerph-19-03357-s001.zip › ijerph-1604955-supplementary.pdf]

Table S1. Multivariate analysis of factors associated with mortality among male and female patients with COVID-19, respectively.

Factors	Multivariate analysis (men)		Multivariate analysis (women)	
	**AOR (95% CI)**	**P value**	**AOR (95% CI)**	**P value**
Age (years)				
18-64	1		1	
≥65	4.48 (2.38-8.45)	<.001	13.61 (4.41-41.98)	<.001
Heart failure	12.90 (2.39-69.66)	0.003	9.73 (0.84-112.45)	0.069
End-stage of renal disease	12.94 (4.21-39.78)	<.001	-	-

COVID-19, coronavirus disease 2019; AOR, adjusted odds ratio; CI, confident interval.
